# The Impact of Myocardial Bridging on the Coronary Functional Test in Patients with Ischaemia with Non-Obstructive Coronary Artery Disease

**DOI:** 10.3390/life12101560

**Published:** 2022-10-08

**Authors:** Hiroki Teragawa, Chikage Oshita, Yuko Uchimura

**Affiliations:** Department of Cardiovascular Medicine, JR Hiroshima Hospital, 3-1-36, Futabanosato, Higashi-ku, Hiroshima 732-0057, Japan

**Keywords:** myocardial bridge, coronary microvascular dysfunction, coronary spasm, spasm provocation test

## Abstract

Background: The possibility of myocardial bridging (MB) causing chest pain has been widely reported; however, the effect of MB on coronary microvessels has not been thoroughly investigated. Therefore, this study evaluated the effects of MB on epicardial coronary artery and coronary microvascular function during coronary angiography (CAG) and coronary function test (CFT) in patients with ischaemia with non-obstructive coronary artery disease (INOCA). Methods: This study included 62 patients with INOCA who underwent CAG and CFT for the left anterior descending coronary artery (LAD) to evaluate chest pain. In the CFT, acetylcholine was first administered intracoronarily in a stepwise manner, followed by chest symptoms, electrocardiographic ST-T changes and CAG. Positive coronary spasm was defined as coronary vasoconstriction of >90% on CAG accompanied by chest symptoms or electrocardiographic ST-T changes. After nitroglycerin administration, CAG was performed to assess MB, which was defined as systolic narrowing of the coronary artery diameter by >20% compared with that in diastole. Coronary flow reserve (CFR) and index of microcirculatory resistance (IMR) were subsequently obtained via transvenous adenosine triphosphate infusion using a pressure wire. Coronary microvascular vasodilatory dysfunction (CMD) was defined as a CFR of <2.0 or an IMR of ≥25 units. Results: Of the 62 patients, 15 (24%) had MB. The patients’ characteristics did not differ between the two groups. Regarding the CAG and CFT results, the presence of coronary spasm in the LAD was higher in the MB (+) group (87%) than in the MB (−) group (53%, *p* = 0.02), whereas the values of CFR (MB (+): 2.7 ± 1.4, MB (−): 2.8 ± 1.1) and IMR (MB (+): 26.9 ± 1.0, MB (−): 30.0 ± 17.3) and the presence of CMD (MB (+): 53%, MB (−): 60%) were similar in the two groups. Conclusions: The findings suggest that MB predisposes patients with INOCA to coronary spasms. Conversely, MBs may have a limited effect on microvessels, particularly in such patients.

## 1. Introduction

Myocardial bridging (MB) is a condition in which the coronary arteries run a segmental intramyocardial course rather than the normal distribution over the epicardial surface of the heart [1,2]. In the majority of individuals with MB, this phenomenon appears to be harmless and has no appreciable impact on the myocardium because coronary blood flow occurs largely during diastole [3]. However, in some cases, acute myocardial infarction, vasospastic angina (VSA), exertional angina and sudden cardiac death have been reported to be caused by MB [4,5,6,7,8,9,10]. Additionally, an increase in reports regarding the relationship between the presence of MB and coronary spasm [11,12,13,14,15,16,17,18] has recently occurred.

Recently, it has been discovered that MB is one of the pathogeneses of ischaemia with non-obstructive coronary artery disease (INOCA) and/or myocardial infarction with non-obstructive coronary arteries (MINOCA) [19,20,21]. Thus, in patients with MB who are documented as having INOCA or MINOCA, the reductions in intracoronary pressure measured using a pressure wire have frequently been reported [19,22,23]. In patients with INOCA or MINOCA, coronary microvascular dilatory dysfunction (CMD) has been increasingly recognised as a cause of prolonged chest pain and has received much attention [24,25]. However, little is known about the relationship between the presence of MB and the occurrence of CMD.

Therefore, in the present study, we performed a comprehensive coronary function test (CFT), including a spasm provocation test (SPT) and a coronary microvascular function test (MVFT), using a pressure wire in patients with INOCA. We also investigated the presence of CMD in patients with MB and INOCA compared that in patients without MB and INOCA.

## 2. Materials and Methods

### 2.1. Study Population

**Figure 1:**
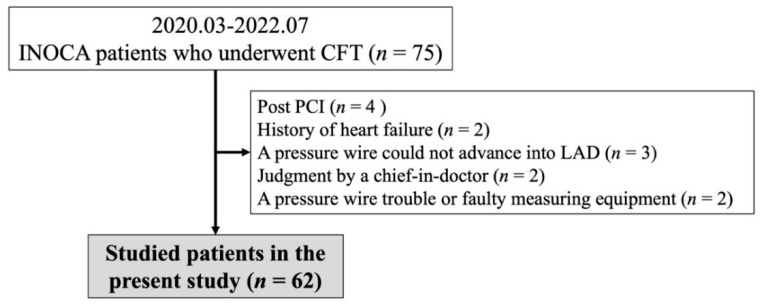
Study flowchart. CFT, coronary function test; INOCA, ischaemia with non-obstructive coronary artery disease; LAD, left anterior descending coronary artery; PCI, percutaneous coronary intervention.

This was a retrospective study of 75 patients with chest pain who had undergone coronary angiography (CAG) and a CFT between March 2020 and July 2022 at our institution. We excluded patients who had moderate coronary stenosis (% stenosis ≥ 50%) or moderate chronic kidney disease (CKD) with an estimated glomerular filtration rate (eGFR) of <45 mL/min/1.73 m^2^ or a history of heart failure or percutaneous coronary intervention (PCI). Thus, four patients who had undergone PCI and two patients with a history of heart failure were excluded. Additionally, two cases for whom a pressure wire or measuring device was not functioning, three patients in whom a guidewire could not be inserted in the left anterior descending coronary artery (LAD) and two patients for whom the doctor-in-charge decided that the vessel for the insertion of the pressure wire would be the right coronary artery (RCA) were excluded. Finally, 62 patients with INOCA were analysed in the present study. The patient selection process and the number of analysed patients who underwent each procedure are presented in Figure 1. The ethics committee of JR Hiroshima Hospital approved this study (2022-18). Informed consent was obtained from all patients.

### 2.2. CFT

The procedures used for the SPT at our institution have been previously described [26,27]. In brief, an SPT was performed following a typical diagnostic CAG using the percutaneous brachial route and a 5-Fr sheath diagnostic Judkins-type catheter. Following the initial CAG, the left coronary artery (LCA) was injected with 50, 100 and 200 μg of acetylcholine (ACh) for 20 s every 3 min. The maximum ACh infusion or the induction of coronary spasms, whichever came first, was followed by immediate CAG. An RCA SPT is then performed without the intracoronary injection of nitroglycerin (NTG) into the LCA if a coronary spasm was produced but resolved spontaneously. After the SPT for the RCA was accomplished, CAG was performed once more following an NTG injection into the LCA. A 0.3-mg intracoronary injection of NTG was administered if ACh infusion into the LCA caused a protracted coronary spasm or if it caused haemodynamic instability. Following spasm induction in the LCA, 20, 50 and 80 μg of ACh was pumped into the RCA for 20 s at 3-min intervals. The maximum ACh infusion or the induction of coronary spasms, whichever came first, was followed by immediate CAG. The last CAG of the RCA was conducted following a 0.3-mg intracoronary infusion of NTG. When the subsequent SPT was negative following the necessary usage of NTG, the result was deemed ‘unable to determine’ (NA).

The techniques used for the MVFT were described in a previous study [28]. The PressureWire X Cabled Guidewire (Abbot Laboratories, Abbot Park, IL, USA) was used with a pressure–temperature sensor tip. The RadiAnalyzerTM Xpress was used to measure the parameters (Abbott Vascular, Santa Clara, CA, USA). The PressureWire was safely advanced in the LAD distally. A thermodilution curve was produced using three injections of 3 mL of saline at room temperature to estimate the resting mean transit time (Tmn). Adenosine triphosphate (160 g/kg/min) was infused intravenously through a peripheral vein to produce hyperaemia. Maximum hyperaemia was used to determine the hyperaemic proximal aortic pressure (Pa), distal arterial pressure (Pd) and hyperaemic Tmn. The lowest average of three successive beats during stable hyperaemia was used to compute the fractional flow reserve (FFR). The ratio of resting Tmn to hyperaemic Tmn was used to compute the coronary flow reserve (CFR). During hyperaemia, the index of microcirculatory resistance (IMR) was computed using the formula Pd × Tmn. Before monitoring these values in each coronary artery, we systematically calibrated the aortic pressure in the catheter and the pressure obtained using the PressureWire to prevent the possibility of pressure drift. Additionally, we verified that there was no pressure drift between the pressure received from the withdrawal of the PressureWire and the aortic pressure.

### 2.3. Definitions of CFT

The procedure for determining the coronary artery diameter was explained in a previous study [29]. We chose segments with spasticity, atherosclerosis and MB for quantitative study. Analysis was performed using the average value obtained from three measurements. The percentage deviation from the initial angiographic data was used to express the changes in coronary artery diameter in response to the ACh and NTG infusions. Atherosclerotic lesions were defined as those that had >20% stenosis. As previously reported [11], we investigated the possibility of MB, which is the presence of a >20% systolic reduction in coronary artery diameter. Measurements for MB were made at the imaging site, where squeezing appeared to be progressing the fastest following NTG injection. Measurements were made of the vessel diameter and angiographic MB length at the most constricted area in both diastole and systole. The difference between the two values was then divided by the vessel’s diastolic diameter and expressed as a percentage squeezing at the MB segment. According to the presence of MB, patients were divided into the following two groups: MB (+) and MB (−).

Coronary spasm was defined as >90% narrowing of the epicardial coronary arteries on angiography during the SPT, the presence of recognisable chest pain and/or ST-segment deviation on electrocardiography (ECG) [27,30]. According to the American Heart Association’s classification, a focal spasm is characterised by a temporary vessel narrowing of >90% that occurs only within the boundaries of a single, isolated coronary segment. A diffuse spasm is described as a >90% diffuse vasoconstriction of the coronary arteries in two contiguous coronary segments [31]. Multi-vessel spasms are defined as coronary spasms that occur in at least two major coronary arteries, and we were unable to determine when the subsequent SPT was negative after an unavoidable use of NTG in one coronary artery. In this study, low, moderate and high doses of ACh were considered to be 50, 100 and 200 µg for the LCA, respectively. Microvascular spasm (MVS) was described as the lack of angiographic coronary spasm associated with typical chest discomfort and ST-T ECG alterations during the SPT [25,32]. IMR values of 25 units or CFR values of 2.0 were used to define CMD [33].

### 2.4. Definitions of Clinical Parameters

We categorised the patients as either active smokers or non-smokers based on their smoking habits. A systolic blood pressure of ≥140 mmHg, a diastolic blood pressure of ≥90 mmHg or taking antihypertensive medication were considered signs of hypertension. Triglycerides, low-density lipoprotein cholesterol, fasting blood sugar, haemoglobin A1C, creatinine, C-reactive protein and N-terminal pro-brain natriuretic peptide levels were assessed. The presence of CKD was determined using accepted criteria [34], and the eGFR (mL/min/1.73 m^2^) was computed using the accepted formula. Both a low-density lipoprotein cholesterol level of ≥120 mg/dL and the use of drugs for dyslipidaemia were considered indicative of dyslipidaemia. A fasting blood sugar level of ≥126 mg/dL, a haemoglobin A1C level of ≥6.5% or the use of anti-diabetic drugs were considered to indicate diabetes mellitus. Using echocardiography, the left ventricular ejection fraction (LVEF) was calculated. The Devereux and Reichek formula [35,36] was used to determine the left ventricular mass index (LVMI). As shown in our previous report [26], a UNEXEF device was used to assess the flow-mediated dilation (FMD) and NTG-mediated dilation (NMD) of the brachial artery; these are objective measurements of the endothelium-dependent and endothelium-non-dependent functions, respectively (UNEX Corp, Nagoya, Japan).

Regarding chest symptoms, the activity of chest pain, when it occurred, was classified into three patterns: resting, exertion and both resting and exertion.

### 2.5. Statistical Analyses

Continuous data were expressed as means with standard deviation. Non-normally distributed data and non-continuous variables were reported as medians with interquartile ranges. Student’s unpaired t-test, Wilcoxon signed-rank test or χ^2^ analysis was used to compare the baseline characteristics of the groups and the results of the CAG and CFT. Spearman’s rank correlation coefficient was used to correlate MB length or %MB with CFR or IMR. Logistic regression analysis was used to determine the presence of VSA and MVD. All statistical analyses were conducted using JMP Ver. 16 (SAS Institute Inc., Cary, NC, USA). A *p*-value of <0.05 was considered significant.

## 3. Results

### 3.1. Patient Characteristics and Clinical Parameters

There were 15 patients (24%) with MB. The patients’ characteristics are shown in Table 1. The average age tended to be higher in the MB (+) group than in the MB (−) group (*p* = 0.06), whereas other factors did not differ between the two groups.

The blood chemical and echographic parameters are shown in Table 2. The value of total cholesterol tended to be higher in the MB (+) group than in the MB (−) group (*p* = 0.08). Regarding the echographic parameters, LVEF and LVMI on echocardiography and FMD and NMD on the brachial artery were not different between the two groups.

### 3.2. VSA-Related Parameters and the Results of CAG and CFT

**Figure 2:**
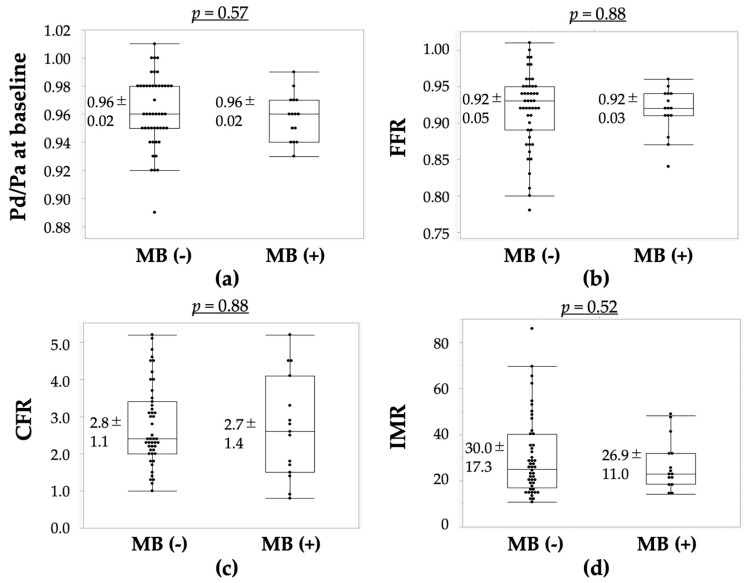
Intracoronary pressure and coronary microvascular dilatory function in the MB (−) and MB (+) groups. (**a**) Values of Pd/Pa at baseline; (**b**) FFR; (**c**) CFR; and (**d**) IMR. There were no significant changes in these values between the MB (−) and MB (+) groups. CFR, coronary flow reserve; FFR, fractional flow reserve; IMR, index of microcirculatory resistance; MB, myocardial bridge; Pa, aortic pressure; Pd, distal pressure.

VSA-related parameters and the results of CAG and CFT are shown in Table 3. There was no difference between the two groups in terms of when chest symptoms occurred during rest, exertion or both resting and exertion. The presence of atherosclerosis on both RCA and LCA, or on LAD, was not different between the two groups. In the MB (+) group, the length of MB was 15.4 ± 5.1 mm and % squeezing at MB segments during systole was 40.1 ± 14.1%. The presence of coronary spasms on LAD was significantly higher in the MB (+) group (87%) than in the MB (−) group (53%, *p* = 0.02). The types of coronary spasms on LAD, such as diffuse and focal spasms, MVS and non-spasm, tended to be different between the two groups (*p* = 0.06); however, with respect to the presence of diffuse and focal spasms and when the epicardial coronary spasm was provoked, it was not different between the two groups: diffuse/focal spasms were 11/14 in the MB (−) group (*n* = 25) and 8/5 in the MB (+) group (*n* = 13, *p* = 0.31). The values of Pd/Pa at baseline, FFR, CFR and IMR were not different between the two groups (Table 3 and Figure 2). Thus, the presence of CMD was 60% in the MB (−) group and 54% in the MB (+) group.

In the MB (+) group, we investigated the relationship between CFR and IMR and the length of MB or % squeezing at MB segments during systole; however, there were no significant relationships (the length vs. CFR: ρ = 0.07, *p* = 0.79; the length vs. IMR: ρ = −0.17, *p* = 0.53; % squeezing vs. CFR: ρ = −0.27, *p* = 0.31; % squeezing vs. IMR: ρ = 0.22, *p* = 0.41).

### 3.3. Factors Affecting VSA

Among 62 patients, 39 patients (63%) had VSA. Regarding the presence of VSA, two significant factors and one factor with trend were observed in the two groups with VSA and without VSA: male gender (54% in the VSA group and 22% in the non-VSA group, *p* = 0.01), MB (33% in the VSA group and 9% in the non-VSA group, *p* = 0.03) and FMD (3.6 ± 2.0% in the VSA group and 4.7% ± 3.0% in the non-VSA group, *p* = 0.08). Logistic regression analysis using three factors showed that MB was a significant factor associated with VSA (Table 4).

## 4. Discussion

In the present study, we investigated the influence of the presence of MB on CFT, including SPT and MVFT, in patients with INOCA. We found that coronary spasm was more frequently observed in patients with MB than in those without MB on SPT. Conversely, coronary microvascular function was similar in patients with MB and without MB; thus, the presence of CMD was similar in the two groups. MB may have a limited effect on coronary microvascular function, whereas it may affect coronary spasm to some extent, in such patients.

In the clinical setting, there are many subjects with MB who have no chest symptoms associated with MB, suggesting that MB may be harmless [3]. However, it has been considered that MB affects the coronary vasculature, leading to the occurrence of chest symptoms, in some patients. In general, regarding the influence of MB on the coronary vasculature, there are the following factors. First, atherosclerosis tends to develop near the proximal side of the MB [37,38,39,40,41]. Second, coronary blood flow is possibly reduced because of the compression of the coronary artery by systolic milking by MB [1,38]. Because of the combination of the presence of atherosclerosis beneath the MB and systolic compression of the coronary artery by MB, it has been reported that FFR is reduced in patients with MB [1]. Third, MB may increase the susceptibility to coronary spasms [11,12,13,14,15,16,17,18].

The exact mechanism involved in this increased susceptibility to coronary spasms remains unclear. One experimental study showed that turbulent flow may increase endothelial cell apoptosis or tumour necrosis factor-alpha-induced endothelial cell activation [42]. Thus, turbulent coronary flow, due to systolic milking by MB, may account for abnormal coronary vasoreactivity at the MB segments. Additionally, optical coherence tomography (OCT) was used by Nishimiya et al. [43] to demonstrate that the adventitial vasa vasorum (VV), which is important for the morphological and functional changes of the coronary vasculature, was easily identifiable at both the proximal and distal segments of the MB, but was less frequently seen within MB segments. Because it has been reported that the increase in adventitial VV is associated with coronary spasm [44], the aforementioned findings may not account for the direct mechanism of coronary spasm at the MB segment itself, but suggest that it may contribute to the occurrence of coronary spasm around MB segments. Because of these possible mechanisms, abnormal vasoreactivity may occur at the MB segments [11,12,45], leading to an increased susceptibility to coronary spasms. The results of the cited study must be discounted because the patients in this study were a group of patients who underwent CFT for suspected coronary spasm and were actually diagnosed with VSA in 63% of the studied patients. Nevertheless, 87% of patients with MB had provoked coronary spasms, which fully confirms the results of previous studies showing an increased susceptibility to coronary spasms in patients with MB [11,12,13,14,15,16,17,18]. Regarding the relationship between coronary spasms and MB, Im et al. [13] showed that coronary spasms in patients with MB was provoked at a low ACh dose, and the spasms were severe, diffuse and long. Conversely, in the present study, the doses of ACh at spasm provocation and the type of coronary spasm, such as diffuse and focal, were not associated with the presence of MB. The small number of studied patients, the differences in patient backgrounds and the possibility that the degree of MB was not as strong, which will be discussed in more detail later, may have contributed to this difference.

The coexistence of CMD may contribute to subsequent prolonged chest symptoms [24,46] and has recently received particular attention [25,47]. In this study, we investigated the effects of MB on coronary microvascular function, which may be the first report of its kind. Our results have demonstrated that the presence of MB did not affect the CFR or IMR, nor did it affect the frequency of CMD. Although the diagnosis of MB is increasingly made using intravascular ultrasound (IVUS) or OCT [21], our study used only coronary angiographic findings. Furthermore, the angiographic definition of MB was 20%, so it is possible that the degree of MB itself was not strong in many cases. Although it has been reported that FFR can be reduced in some patients with MB [1], there were no patients with FFR <0.8 in the MB group in the present study. It has been reported that atherosclerosis tends to develop proximal to MB [37,38,39,40,41]; however, the frequency of atherosclerosis was not different between patients with MB and those without MB in the present study. These findings may suggest that the degree of MB in the present study may not have been strong. Nevertheless, even in the small number of cases we investigated, a correlation between MB length and % squeezing with CFR and IMR was not detected. These results suggest that MB, even if it is strong in degree, does not affect coronary microvascular function. Further studies of larger numbers of cases and multicentre registries are needed to confirm our results.

There were several study limitations to the present study. First, this study was retrospective and examined only a small number of studied patients at a single institution. Second, as mentioned above, the diagnosis of MB was made only via CAG and not via intravascular imaging, such as IVUS or OCT. Additionally, the definition of MB was set at 20%, which may have included MBs of less intense degree overall. However, in our previous study, the MB (−) group did not exceed 15% [11]; thus, this definition may be valid. Finally, in the present study, we only examined LAD, and in fact, there were no cases of MB other than LAD in our studied patients. However, it has been reported that MBs can be found in coronary arteries other than the LAD [48], and it is unclear whether the present findings are applicable to such vessels.

## 5. Conclusions

We demonstrated that MB predisposes patients with INOCA to coronary spasms. Conversely, MBs may have a limited effect on microvessels, particularly in such patients. Further studies, including larger numbers of patients and at multicentre registries, are needed to confirm these findings.

## Figures and Tables

**Table 1:** Patients’ characteristics for the MB (−) and MB (+) groups.

		MB (−) Group	MB (+) Group	*p* Value
No (%)		47 (76)	15 (24)	
Age (years)		63 ± 14	71 ± 15	0.06
Male/Female		17/30	9/6	0.1
Body mass index		24.1 ± 4.4	24.4 ± 3.7	0.83
Coronary risk factors (%)				
	Current smoker	7 (15)	1 (7)	0.41
	Hypertension	30 (64)	8 (53)	0.47
	Dyslipidaemia	25 (53)	5 (33)	0.18
	Diabetes mellitus	7 (15)	1 (7)	0.41
Family history of CAD (%)		7 (15)	3 (20)	0.64
CKD (%)		11 (23)	2 (13)	0.4
Taking statins (%)		23 (49)	4 (27)	0.13

Numbers were expressed as numbers (percentage), and values were expressed as the mean with standard deviation. CAD, coronary artery disease; CKD, chronic kidney disease; MB, myocardial bridge; No., numbers.

**Table 2:** Blood chemical and echographic parameters for the MB (−) and MB (+) groups.

		MB (−) Group	MB (+) Group	*p* Value
Blood chemical parameters				
	Total cholesterol (mg/dL)	192 ± 33	176 ± 29	0.08
	Triglyceride (mg/dL)	118 ± 71	95 ± 27	0.22
	HDL-cholesterol (mg/dL)	61 ± 15	60 ± 13	0.9
	LDL-cholesterol (mg/dL)	109 ± 29	97 ± 28	0.18
	Fasting blood glucose (mg/dL)	103 ± 17	105 ± 13	0.72
	Haemoglobin A1c (%)	6.0 ± 0.6	5.9 ± 0.3	0.7
	C-reactive protein (mg/dL)	0.06 (0.03, 0.10)	0.03 (0.02, 0.09)	0.33
	eGFR (mL/min/1.73 m^2^)	69.8 ± 13.1	6.8.3 ± 15.4	0.71
	NT proBNP (pg/mL)	71 (36, 150)	131 (64, 214)	0.17
Echographic parameters				
	LVEF (%)	66 ± 6	65 ± 7	0.4
	LVMI (g/m^2^)	83 ± 21	79 ± 20	0.48
	FMD (%)	4.2 ± 2.5	3.5 ± 2.0	0.33
	NMD (%)	14.5 ± 7.0	13.5 ± 3.1	0.59

Values were expressed as the mean with standard deviation or as the median with interquartile ranges, if appropriate. NT proBNP, N-terminal pro-brain natriuretic peptide; eGFR, estimated glomerular filtration ratio; FMD, flow-mediated dilation; HDL, high-density lipoprotein; LDL, low-density lipoprotein; LVEF, left ventricular ejection fraction; LVMI, left ventricular mass index; MB, myocardial bridge; NMD, nitroglycerin-mediated dilation.

**Table 3:** VSA-related parameters and results of CAG and CFT for the MB (−) and MB (+) groups.

		MB (−) Group	MB (+) Group	*p* Value
Chest symptoms				
	At rest/exertion/both	43/0/4	12/1/2	0.17
CAG				
	Atherosclerosis of both coronary arteries (%)	23 (49)	7 (47)	0.88
	Atherosclerosis on LAD (%)	14 (30)	7 (47)	0.23
	Length of MB (mm)		15.4 ± 5.1	
	% Squeezing of MB segments		40.1 ± 14.1	
CFT				
	Presence of coronary spasm (%)	26 (55)	13 (87)	0.03
	Presence of coronary spasm in LAD (%)	25 (53)	13 (87)	0.02
	Types of coronary spasm (diffuse/focal/MVS/no-spasms)	14/11/10/12	5/8/2/0	0.06
	Presence of multi-vessel spasms (%, n)	13 (52, *n* = 25)	7 (58, *n* = 12)	0.72
	The dose of ACh at spasm provocation (Low/moderate/high, n)	2/19/4 (n = 25)	2/7/4 (n = 13)	0.38
	Pd/Pa at baseline	0.96 ± 0.02	0.96 ± 0.02	0.57
	FFR	0.92 ± 0.05	0.92 ± 0.03	0.88
	CFR	2.8 ± 1.1	2.7 ± 1.4	0.88
	IMR	30.0 ± 17.3	26.9 ± 11.0	0.52
	Presence of CMD	28 (60)	8 (53)	0.67

Numbers were expressed as numbers (percentage), and values were expressed as the mean with standard deviation. Ach, acetylcholine; CAG, coronary angiography; CFR, coronary flow reserve; CFT, coronary function test; CMD, coronary microvascular dilatory dysfunction; FFR, fractional flow reserve; IMR, index of microcirculatory resistance; LAD, left anterior descending coronary artery; MB, myocardial bridge; MVS, microvascular spasm; VSA, vasospastic angina.

**Table 4:** Logistic regression analyses for factors associated with the presence of VSA.

Factors	Odds Ratio	*p* Value
Presence of MB	5.15	0.02
Male gender	2.73	0.09
%FMD	1.31	0.25

R^2^ = 0.16. FMD, flow-mediated dilation; MB, myocardial bridge; VSA, vasospastic angina.

## Data Availability

Not applicable.
